# On the Performance Evaluation of Commercial SAW Resonators by Means of a Direct and Reliable Equivalent-Circuit Extraction

**DOI:** 10.3390/mi12030303

**Published:** 2021-03-14

**Authors:** Giovanni Gugliandolo, Zlatica Marinković, Giuseppe Campobello, Giovanni Crupi, Nicola Donato

**Affiliations:** 1 MIFT Department, University of Messina, 98166 Messina, Italy; giovanni.gugliandolo@unime.it; 2 Faculty of Electronic Engineering, University of Niš, 18000 Niš, Serbia; zlatica.marinkovic@elfak.ni.ac.rs; 3 Engineering Department, University of Messina, 98166 Messina, Italy; gcampobello@unime.it; 4 BIOMORF Department, University of Messina, 98100 Messina, Italy; crupig@unime.it; 5 INSTM Consorzio Interuniversitario Nazionale per la Scienza e Tecnologia dei Materiali, 50121 Florence, Italy

**Keywords:** circuit modeling, metrological evaluation, resonators, scattering parameter measurements, surface acoustic waves

## Abstract

Nowadays, surface acoustic wave (SAW) resonators are attracting growing attention, owing to their widespread applications in various engineering fields, such as electronic, telecommunication, automotive, chemical, and biomedical engineering. A thorough assessment of SAW performance is a key task for bridging the gap between commercial SAW devices and practical applications. To contribute to the accomplishment of this crucial task, the present paper reports the findings of a new comparative study that is based on the performance evaluation of different commercial SAW resonators by using scattering (*S*-) parameter measurements coupled with a Lorentzian fitting and an accurate modelling technique for the straightforward extraction of a lumped-element equivalent-circuit representation. The developed investigation thus provides ease and reliability when choosing the appropriate commercial device, depending on the requirements and constraints of the given sensing application. This paper deals with the performance evaluation of commercial surface acoustic wave (SAW) resonators by means of scattering (*S*-) parameter measurements and an equivalent-circuit model extracted using a reliable modeling procedure. The studied devices are four TO-39 packaged two-port resonators with different nominal operating frequencies: 418.05, 423.22, 433.92, and 915 MHz. The *S*-parameter characterization was performed locally around the resonant frequencies of the tested SAW resonators by using an 8753ES Agilent vector network analyzer (VNA) and a home-made calibration kit. The reported measurement-based study has allowed for the development of a comprehensive and detailed comparative analysis of the performance of the investigated SAW devices. The characterization and modelling procedures are fully automated with a user-friendly graphical user interface (GUI) developed in the Python environment, thereby making the experimental analysis faster and more efficient.

## 1. Introduction

Surface acoustic wave (SAW) devices are key electronic components, which were firstly described by Lord Rayleigh in 1885 [1]. They are able to convert electrical energy into mechanical waves on the basis of the inverse piezoelectric effect. Basically, SAW devices are realized using an interdigital transducer (IDT) on a piezoelectric crystal substrate, such as quartz or lithium niobate (LiNbO_3_) [2]. When excited by an AC voltage coming from an external voltage source or RF signal, the IDT provides, as a consequence of the inverse piezoelectric effect, the electric field necessary to displace the substrate and thus to form an acoustic wave on the piezoelectric crystal substrate. Variations of physical parameters change the characteristics of acoustic waves, such as velocity, phase shift, and intensity. Therefore, by monitoring acoustic wave characteristics (or the resonant frequency of the device), these environmental properties can be measured. In particular, by exploiting the sensitivity of piezoelectric substrate materials to physical parameters, it is possible to use SAW devices as temperature, humidity, and strain sensors [3,4], as well as for the detection of gases [5] and liquids [6]. Moreover, SAW devices are largely employed in telecommunications with more than three billion SAW components manufactured every year and used as band pass filters and resonators in radio receivers of mobile cell phones, base stations, and RF front ends [7,8,9,10,11,12,13]. Finally, they are employed in many other different areas, such as automotive [14] and bioengineering applications, where they are used, for instance, as microfluidic devices [15,16,17] by exploiting the fact that, by inducing an electric field and generating a mechanical wave, it is possible to pump and drive liquids. The main advantages of SAW-based sensors as opposed to other kinds of sensors are their low cost, small size, high-temperature stability, and the possibility to be used as wireless passive sensors [18]. Moreover, high precision can be obtained considering that shifts in frequency are far easier to measure [19,20] and less noise-sensitive than perturbations in voltage or current signals [21].

In this paper, we present a measurement-based performance evaluation of four commercial TO-39 packaged two-port SAW resonators working in a frequency range spanning from 418.05 to 915 MHz. The analyzed devices are experimentally characterized by performing measurements of the scattering (*S-*) parameters that are transformed into the corresponding admittance (*Y-*) form, which is more appropriate for analysis and modeling purposes. The values of the resonant frequency (*f_r_*), the quality factor (*Q_r_*), and the amplitude of the resonant peak have been estimated using a Lorentzian fitting of the measured *Re*(*Y*_11_). Next, an equivalent-circuit model is extracted for the tested devices directly from the measured data sets by exploiting the observed resonance without using optimization and/or tuning. The extracted model is able to faithfully reproduce the measurements locally around the resonant frequency for all of the different tested SAW devices, thereby confirming the accuracy and reliability of the used modeling technique. The achieved values of *f_r_* and *Q_r_*, as well as the extracted circuit elements, are reported and discussed in detail, thus enabling a quantitative evaluation and comparative analysis of the performance of the four different SAW devices under consideration in the present study. The significance of this study lies in the fact that, although the achieved findings can strongly depend on the specific studied devices, the investigation methodology, based on the equivalent-circuit extraction from *S*-parameter measurements, is technology independent and straightforwardly applicable to different commercial SAW devices, allowing for a deeper analysis of the device behavior and appropriate selection of the device according to the requirements of the given sensing application.

## 2. Characterization and Theoretical Analysis

**Figure 1:**
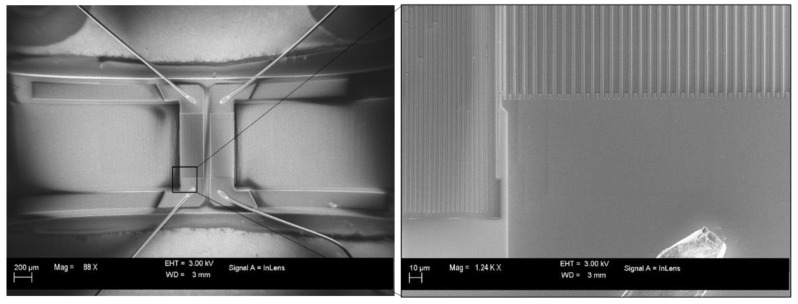
Two SEM micrographs of an R2630 SAW resonator working at 418.05 MHz: (**left side**) overview and (**right side**) particular magnification.

The tested devices are four commercial TO-39 packaged SAW resonators, which have the manufacturer codes R2630 (Siemens Matsushita, Munich, Germany), SAR423.22MDA (Murata, Kyoto, Japan), RP1308 (Murata), and RP1094 (RF Monolithics Murata) and nominal resonant frequencies of 418.05, 423.22, 433.92, and 915 MHz, respectively [22,23,24,25]. Figure 1 shows two scanning electron microscope (SEM) micrographs of an R2630 SAW device with a resonant frequency of 418.05 MHz. The metallic TO-39 package was carefully removed in order to expose the resonator structure.

The *S*-parameters of the four tested SAWs were measured using an 8753ES Agilent vector network analyzer (VNA). A full two-port calibration was performed using a short-open-load-through (SOLT) technique based on a custom calibration kit [26].

**Figure 2:**
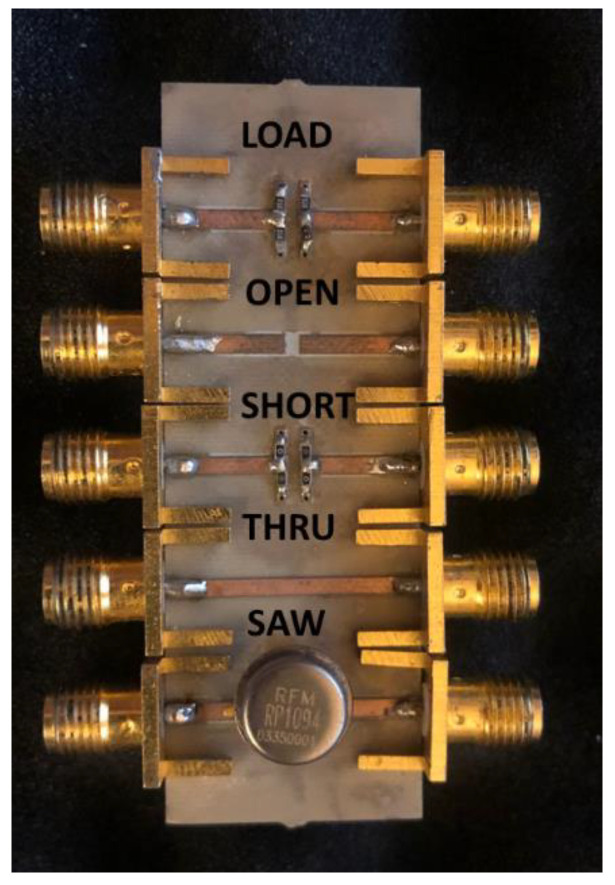
Photo of the developed custom SAW test fixture with on board SOLT calibration kit.

**Figure 3:**
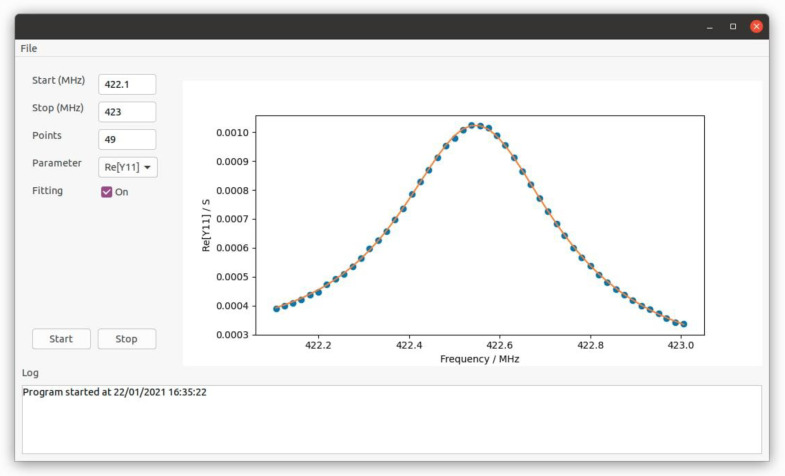
Illustration of the GUI developed in Python environment.

Figure 2 shows the developed test fixture with a custom calibration kit directly on board, which was developed on Arlon substrate by means of a Protomat S103 rapid PCB (by LPKF, Garbsen, Germany) prototyping machine. In order to perform the measurement, the board is connected to the VNA that is controlled remotely from a personal computer via IEEE 488.2 GPIB interface. Both the characterization and the equivalent circuit model extraction are handled by a graphical user interface (GUI) developed in Python environment (see Figure 3).

The remainder of this section is divided into two subsections. The former is focused on the Lorentzian fitting of the measured data, while the latter is devoted to the equivalent-circuit modeling.

### 2.1. Lorentzian Fitting

Resonant frequency, quality factor, and amplitude of the resonant peak in *Re*(*Y*_11_) were evaluated after a Lorentzian fitting of the acquired data points. This strategy is usually employed for a more accurate determination of these parameters [27,28]. In this work, the Lorentzian function in the form reported that the following equation was used as a reference function for the fitting:

(1) $$Re\left(Y_{11}\right)=\frac{a_{0}}{\pi }\cdot \frac{\frac{1}{2}G}{{\left(f-f_{r}\right)}^{2}+{\left(\frac{1}{2}G\right)}^{2}}$$

where *f* is the frequency, *a*_0_ is a real coefficient, and *G* is the full width at half maximum.

**Figure 4:**
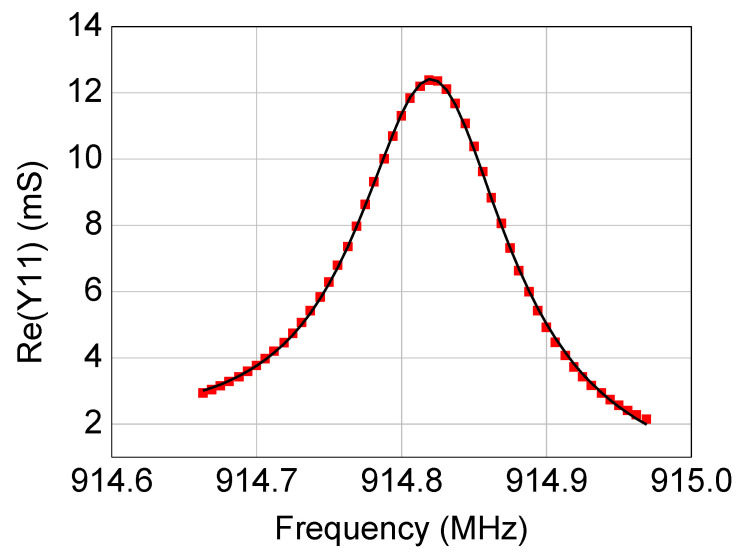
Comparison between (red symbols) measurements and (black line) Lorentzian fitting of the frequency-dependent behavior of *Re*(*Y*_11_) for the 915 MHz SAW resonator.

Moreover, in order to further improve the fitting procedure, a first-order polynomial function was selected to model the effects of background in the spectrum measurements [29]. Since the acquired spectrum consists of a contribution of the resonance plus a background signal [29], *Re*(*Y*_11_) was modeled as a Lorentzian function plus a polynomial function as reported in the following equation:

(2) $$Re\left(Y_{11}\right)=\frac{c_{0}}{\pi }\cdot \frac{\frac{1}{2}G}{{\left(f-f_{r}\right)}^{2}+{\left(\frac{1}{2}G\right)}^{2}}+\overset{N}{\underset{n=0}{\sum }}a_{n}f^{n}$$

where *f* is the frequency, *c*_0_ and *α_n_* are real coefficients, *G* is the full width at half maximum of the Lorentzian function, and *N* is the degree of the polynomial function used to model the background signal. In this case, *N* = 1 was enough for a good fitting. The fitting engine was developed in Python and is based on the Levenberg–Marquardt algorithm. As an illustrative example, Figure 4 shows the comparison between acquired data points and the fitted function of *Re*(*Y*_11_) for the SAW resonator with a resonant frequency of 915 MHz.

### 2.2. Equivalent Circuit Extraction

**Figure 5:**
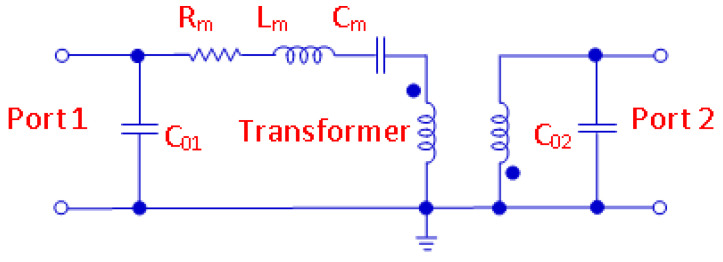
Lumped-element equivalent-circuit model for the investigated two-port SAW resonators.

The two-port equivalent-circuit model employed for the tested SAW resonators is depicted in Figure 5 [30,31,32]. This circuit is able to reproduce the SAW resonator performance near the resonant frequency. The elements *C*_01_ and *C*_02_ are, respectively, the input and output shunt static (non-motional) capacitances between either pin 1 and ground or pin 2 and ground, including the case parasitic capacitance. The elements *C_m_*, *L_m_*, and *R_m_* are the motional parameters modeling the contributions of elasticity, inertia, and damping, respectively. The ideal transformer is associated with the conversion between mechanical and electrical energy.

This model shows that the resonance occurs when the inductive and the capacitive reactance of the motional series RLC network cancel each other out, leading to the following definition of the *Y*-parameters, *f_r_*, and *Q_r_* in terms of the equivalent-circuit elements:

(3) $$Y_{11r}=j\omega_{r}C_{01}+\frac{1}{R_{m}}\;\;\;\;\;\;\;\;\;$$

(4) $$Y_{21r}=Y_{12r}=\frac{1}{R_{m}}\;\;$$

(5) $$Y_{22r}=j\omega_{r}C_{02}+\frac{1}{R_{m}}\;\;\;$$

(6) $$f_{r}=\frac{1}{2\pi \sqrt{L_{m}C_{m}}}\;\;$$

(7) $$Q_{r}=\frac{2\pi f_{r}L_{m}}{R_{m}}=\frac{1}{2\pi f_{r}C_{m}R_{m}}=\frac{1}{R_{m}}\sqrt{\frac{L_{m}}{C_{m}}}\;$$

Therefore, from Equations (3)–(7), we obtain that the five equivalent-circuit elements can be straightforwardly determined as reported below:

(8) $$R_{m}=\frac{1}{Re\left(Y_{11r}\right)}\;\;$$

(9) $$C_{01}=\frac{Im\left(Y_{11r}\right)}{2\pi f_{r}}$$

(10) $$C_{02}=\frac{Im\left(Y_{22r}\right)}{2\pi f_{r}}$$

(11) $$L_{m}=\frac{Q_{r}R_{m}}{2\pi f_{r}}\;\;$$

(12) $$C_{m}=\frac{1}{{\left(2\pi f_{r}\right)}^{2}L_{m}}\;\;$$

An important figure of merit to evaluate resonator performance is given by the product between *f_r_* and *Q_r_*. Equation (7) shows that this quantity can be straightforwardly defined in terms of equivalent-circuit elements as follows:

(13) $$f_{r}Q_{r}=\frac{1}{2\pi R_{m}C_{m}}\;\;$$

## 3. Experimental Results and Discussion

**Figure 6:**
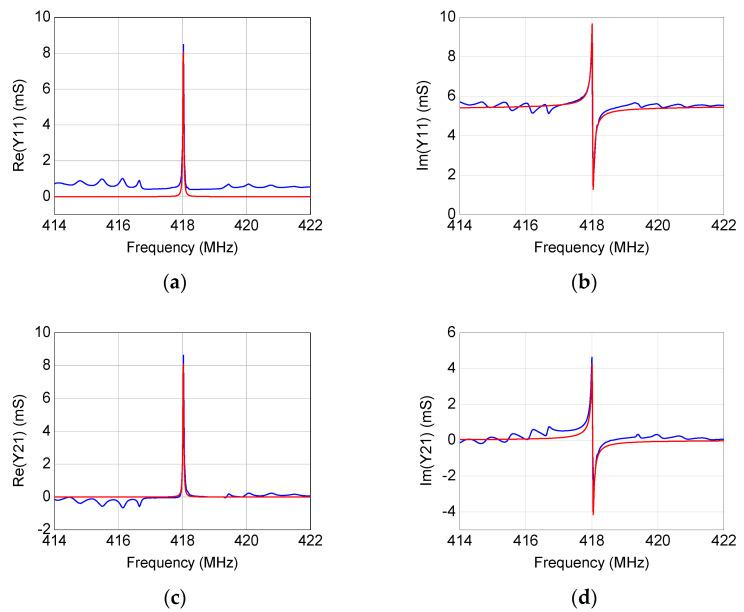

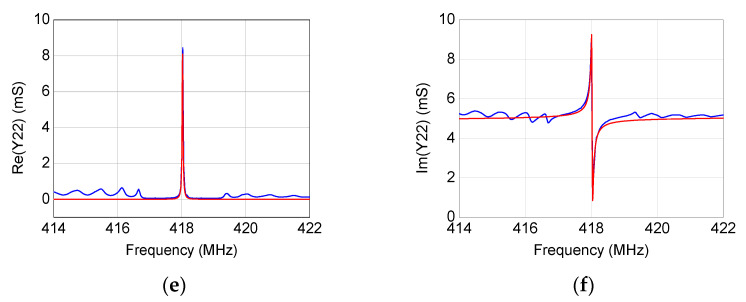
Comparison between (blue) measured and (red) simulated frequency-dependent behavior of (**a**,**b**) *Y*_11_, (**c**,**d**) *Y*_21_, and (**e**,**f**) *Y*_22_ for the 418.05 MHz SAW resonator.

**Figure 7:**
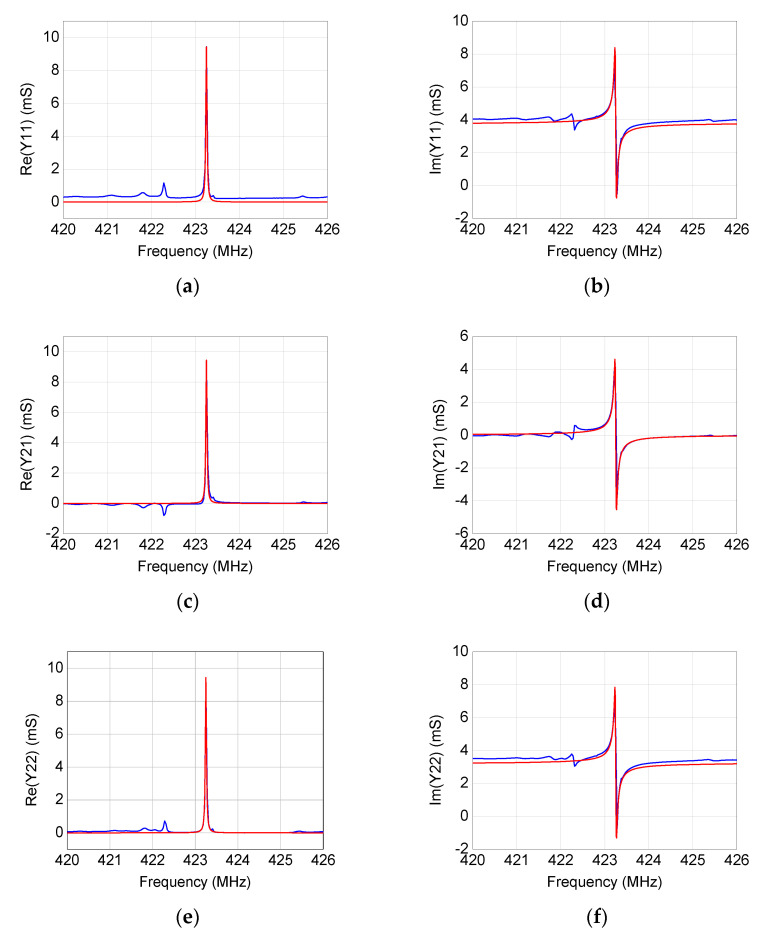
Comparison between (blue) measured and (red) simulated frequency-dependent behavior of (**a**,**b**) *Y*_11_, (**c**,**d**) *Y*_21_, and (**e**,**f**) *Y*_22_ for the 423.22 MHz SAW resonator.

**Figure 8:**
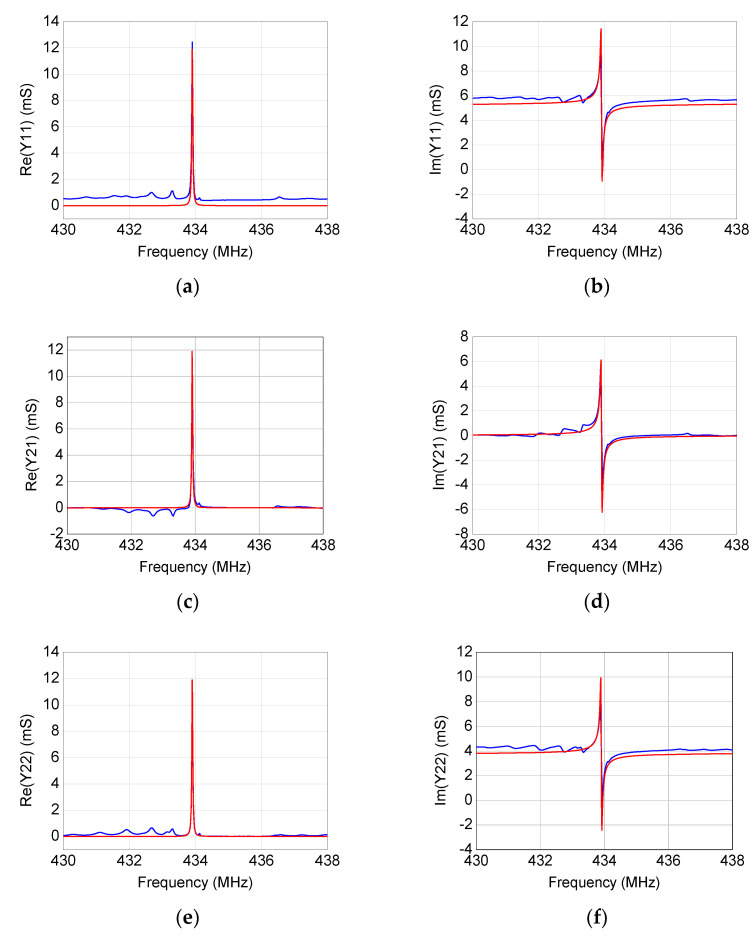
Comparison between (blue) measured and (red) simulated frequency-dependent behavior of (**a**,**b**) *Y*_11_, (**c**,**d**) *Y*_21_, and (**e**,**f**) *Y*_22_ for the 433.92 MHz SAW resonator.

**Figure 9:**
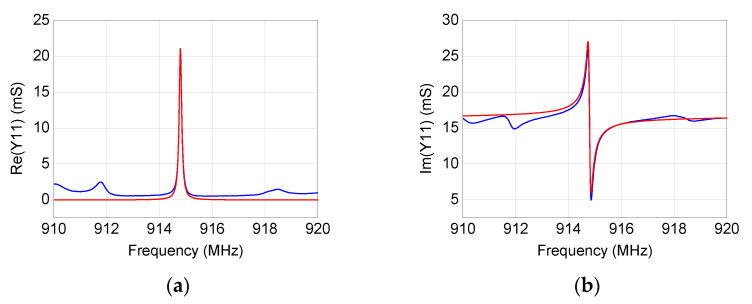

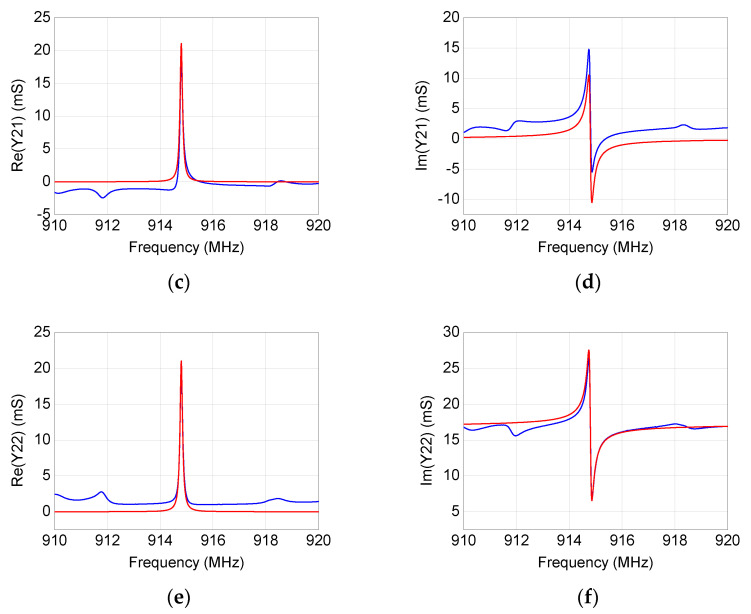
Comparison between (blue) measured and (red) simulated frequency-dependent behavior of (**a**,**b**) *Y*_11_, (**c**,**d**) *Y*_21_, and (**e**,**f**) *Y*_22_ for the 915 MHz SAW resonator.

By using the modeling procedure described in the previous section, the equivalent-circuit model has been extracted for the four studied SAW resonators. As illustrated in Figure 6, Figure 7, Figure 8 and Figure 9, the accuracy and reliability of this extraction technique is confirmed by the achieved good agreement between measured and simulated admittance parameters for all four tested devices. As expected, the extracted model is able to faithfully reproduce the measurements mostly locally near the resonance, whereas some deviations can be observed when the operating frequency departs from the resonant frequency. Although including more equivalent-circuit elements might lead to a better fit over a broader frequency range, the reported model offers a good trade-off between model complexity and prediction accuracy, enabling the use of the extracted model for investigating the resonance.

Table 1 lists the resonant frequency and the quality factor, which were evaluated from the resonance in the measured *Re*(*Y*_11_) by using the Lorentzian fitting, as well as their product and the extracted values of the circuit elements, which were obtained with the illustrated direct modeling procedure.

As can be observed, the values of the resonant frequencies are very close to those specified in the datasheets of the tested components. The quality-factor is roughly the same by keeping *f_r_* at about 400 MHz, whereas its values reduce to almost half when *f_r_* is roughly doubled to slightly more than 900 MHz. Therefore, the product *f_r_Q_r_* of the tested SAWs remains roughly constant (about 5–7 × 10^6^ MHz), exhibiting the highest value when considering the SAW with the highest *f_r_*. The observation that the obtained values of *f_r_Q_r_* are reasonably similar can be linked to the fact that all of the tested SAWs have a piezoelectric crystal substrate made of quartz, which is widely used as a substrate material for commercial SAW devices because of its low cost and remarkable physical properties [33,34,35]. The input shunt static capacitance is about 2 pF and exhibits a significant increase to about 3 pF for the SAW with the highest *f_r_*, whereas the output shunt static capacitance is slightly different than the input one to model the observed differences between *Y*_11_ and *Y*_22_. By increasing *f_r_*, the motional resistance decreases, which turns into a larger amplitude of the resonant peak in *Re*(*Y*_11_). Similar values of the motional inductance and capacitance are observed for the three SAW resonators with *f_r_* at about 400 MHz, whereas the device with the highest *f_r_* shows a small increase in *C_m_* and a remarkable decrease in *L_m_*. The latter is responsible for the much higher value of *f_r_*.

Finally, it should be noted that, when having access to highly advanced and expensive manufacturing facilities, finite element method (FEM) simulations can be used to develop a physically based model for SAW design optimization [36,37,38,39], in order to meet the specific application requirements. On the other hand, when using commercial devices, the user has access to only the information available in the datasheets. In this case, the extraction and analysis of a measurement-based equivalent-circuit model is an essential task when selecting a commercial device for sensing applications, since the equivalent-circuit model allows for building the bridge between SAW design and practical applications.

## 4. Conclusions

A quantitative evaluation and comparative analysis of the performance of four commercial surface acoustic wave resonators with different resonant frequencies have been reported. To achieve this goal, scattering parameter measurements have been carried out in conjunction with Lorentzian fitting and model extraction. The former has enabled an excellent reproduction of the resonance in the real part of *Y*_11_, whereas the equivalent-circuit model has allowed for obtaining a good agreement between measured and simulated admittance parameters as a function of the frequency, which is local to the resonance.

The obtained values of the resonant frequency, quality factor, and lumped equivalent-circuit elements have been analyzed and discussed, in order to achieve a quantitative evaluation and comparative analysis of the performance of the studied four surface acoustic wave resonators with different resonant frequencies.

## Figures and Tables

**Table 1:** Values of the resonant frequency, quality factor, their product, and equivalent-circuit elements for the four studied SAW resonators.

SAW Device (Nominal Frequency)	f_r_ (MHz)	Q_r_	f_r_Q_r_ (MHz)	C_01_ (pF)	C_02_ (pF)	R_m_ (Ω)	L_m_ (μH)	C_m_ (fF)
R2630 (418.05 MHz)	418.03	12,514	5,231,227	2.07	1.92	117.9	561.8	0.26
SAR423.22 MDA (423.22 MHz)	423.25	13,649	5,776,939	1.42	1.23	105.8	542.9	0.26
RP1308 (433.92 MHz)	433.90	13,439	5,831,182	1.94	1.41	80.3	395.8	0.34
RP1094 (915 MHz)	914.80	7988	7,307,422	2.88	2.97	47.5	66.0	0.46
